# Psychosocial health of patients receiving orthopaedic treatment in northern Tanzania: A cross-sectional study

**DOI:** 10.1016/j.amsu.2019.10.020

**Published:** 2019-11-02

**Authors:** Joy E. Obayemi, Elizabeth B. Card, Octavian Shirima, Honest Massawe, Faiton Mandari, Anthony Pallangyo, Rogers Temu, Ajay Premkumar, Neil P. Sheth

**Affiliations:** a Perelman School of Medicine, University of Pennsylvania, 3400 Civic Center Boulevard, Philadelphia, PA, 19104, USA; b Department of Orthopaedic Surgery, Kilimanjaro Christian Medical Centre, PO Box 3010, Moshi, Tanzania; c Hospital for Special Surgery, 535 E 70th Street, New York, NY, 10021, USA; d Department of Orthopaedic Surgery, University of Pennsylvania, 800 Spruce Street, Philadelphia, PA, 19107, USA

**Keywords:** Global surgery, Orthopaedics, LMIC, Psychosocial health

## Abstract

**Background:**

Patients with musculoskeletal injuries in Sub-Saharan Africa often receive prolonged inpatient treatment due to limited access to surgical care. Little is known regarding the psychosocial impact of prolonged conservative treatment for orthopaedic injuries, which may add to disability and preclude rehabilitation.

**Methods:**

A cross-sectional, questionnaire study was conducted to characterize the psychosocial health of orthopaedic inpatients at a tertiary hospital in Moshi, Tanzania. Three validated surveys assessing coping strategies, functional social support, and symptoms of depression were orally administered to all orthopaedic patients with a length of stay (LOS) ≥ 6 days by a Tanzanian orthopaedic specialist.

**Results:**

Fifty-nine patient surveys were completed, and revealed 92% (54) of patients were more likely to utilize more adaptive than maladaptive coping strategies. Patients with chest or spinal column injuries were more likely to use maladaptive coping strategies (p = 0·027). Patients with head injuries had more social support compared to others (p = 0·009). Lack of insurance, limited education, and rural origins were associated with less functional social support, although this finding did not reach statistical significance. 23·7% (14) of patients had symptoms consistent with mild depression, 33·9% (20) with moderate depression, and 3·4% (2) with moderately-severe depression. LOS was the only significant predictor for depression severity.

**Conclusions:**

61% (36) of orthopaedic inpatients exhibited depressive symptoms, indicating that the psychosocial health in this population is sub-optimal. Mental health is a crucial element of successful orthopaedic care. Access to timely surgical care would greatly decrease LOS, the most prominent predictor of depressive symptom severity.

## Highlights

• The majority of orthopaedic patients were found to have symptoms of depression.
• Length of stay was the only significant predictor of depression symptom severity.
• Patients with torso injuries tended to use more maladaptive coping strategies.

## 1 Introduction

Musculoskeletal injuries, often resulting from road traffic crashes (RTCs), continue to impose a significant burden on the health care systems of resource-limited countries such as Tanzania. It is estimated that these injuries rank ninth globally among the leading causes of disability adjusted life years lost [1]. A 2016 study conducted in Tanzania demonstrated that approximately 70% of the victims of RTCs reported were between 18 and 45 years old [2]. The resulting disability from RTCs in this population often leaves working-age men and women with a diminished capacity to care for their families and decreased ability to contribute meaningfully to the nation's developing economy. Given these detrimental effects, any improvements to the treatment of musculoskeletal injuries would provide an enormous benefit to the thousands of individuals injured by RTCs each year in Tanzania and the millions of individuals affected worldwide [2,3]. One aspect of injury treatment and rehabilitation that requires further investigation is the way in which we address the mental health needs of this patient population.

Mental health services have historically been under-utilized by orthopaedic specialists and therapists despite evidence that musculoskeletal injuries result in significant psychopathology [4,5]. Van Delft-Schreurs et al. [6] found that among severely injured patients, long-term physical complaints were mainly reported by those with concomitant psychological complaints. It is well known that patients who develop psychological disorders such as anxiety, depression, or post-traumatic stress disorder (PTSD) are more likely to manifest physical discomfort [6]. Additional studies have shown that psychiatric symptoms accounted for a larger proportion of the disability found 12 months following a traumatic injury than both physical factors and pain severity [7]. These findings all support the fact that a patient's mental health can directly impact their physical experience of an injury and their ability to achieve successful rehabilitation.

Recovery from musculoskeletal injuries can often take an extended period of time, especially in Sub-Saharan Africa. Conservative treatments are very common in low-middle income countries (LMICs) such as Tanzania due to a persistent lack of resources needed to provide more expedient surgical intervention [3]. In cases where fractures are treated definitively with skeletal traction for six to eight weeks, patients are immobilized throughout their hospital admission [3]. A previous study conducted in a tertiary hospital in northern Tanzania – the same hospital as the one in our study – found that 40% (n = 213) of patients with femoral fractures were treated conservatively with traction, thereby illustrating that long periods of immobilization are common practice in this setting [8]. While some work has been done on PTSD that can result from a traumatic accident, little has been done to evaluate the prevalence of other psychiatric disorders that can develop or be exacerbated by difficult treatment courses [[9], [10]]. Furthermore, markers of general psychosocial health and coping have also been relatively unexplored in this context.

The objectives of this study were to evaluate the psychosocial health of patients receiving orthopaedic treatment for musculoskeletal injuries in Northern Tanzania. Three distinct aspects of psychosocial health were analyzed: the severity and prevalence of depression symptoms, mental and emotional coping mechanisms utilized, and the degree of functional social support experienced by each patient.

## 2 Methods

### 2.1 Setting

This study received local Institutional Review Board approval and ethics committee approval from the Kilimanjaro Christian Medical College (Research Ethical Clearance No 2220). In this cross-sectional analysis, we surveyed patients receiving treatment at the Kilimanjaro Christian Medical Center (KCMC) orthopaedic department over a two-month period (June 1st to July 31st, 2017). Inclusion criteria included age >15-years-old and treatment duration of ≥6 days.

### 2.2 Survey protocol

A survey was created for the purposes of this study by combining and translating (when necessary) three validated surveys that pertain to the mental health of patients. All translation was completed by a Tanzanian translator fluent in both English and Swahili, the official language of Tanzania. These translations were then tested by three native Tanzanians for readability and comprehensibility. Final edits were made by a Tanzanian orthopaedic specialist to ensure that the content remained clear and appropriate for the Northern Tanzanian context. After obtaining informed consent, the same Tanzanian orthopaedic specialist orally administered the combined survey to all orthopaedic patients who met the inclusion criteria in the allotted time frame. A total of 59 surveys were fully completed and collected for analysis (100% response rate).

An abbreviated version of the COPE inventory, written and validated at the University of Miami, was used to assess patient coping strategies [11,12]. 14 coping strategies (Table 1) were probed using one or two statements. For each statement, the patient was asked to rate on a scale of 1–4 (1 = I haven't been doing this at all, 4 = I've been doing this a lot) how often the statement applied to their actions (see Appendix A and B). These strategies were further categorized as maladaptive or adaptive by our research team. This categorization was informed by psychological evidence indicating that coping strategies that “both reduce stress and help resolve the problem” allow individuals to be optimally adjusted [13]. Based on responses, each study participant was given a maladaptive and adaptive coping score.

**Table 1:** Adaptive and maladaptive coping strategies measured in survey questionnaire.

Adaptive Coping Strategies	Maladaptive Coping Strategies
Active Coping	Denial
Positive reframing	Self-distraction
Planning	Substance abuse
Acceptance	Behavioral disengagement
Religion	Venting
Use of emotional support	Self-blame
Use of instrumental support	
Humor	

To assess functional social support, a modified version of the Duke-UNC Functional Social Support Questionnaire (FSSQ) was administered [14]. This survey assesses the quality of the patients’ social relationships by evaluating confidant and affective support (see Appendix A). Patients are asked on a scale of 1–4 how much a given statement applies to their current situation and theses values are totaled. While validated versions of the FSSQ have been administered in several languages, no validated Swahili translation has been documented, requiring our team to develop a version independently [[15], [16], [17]]. However, there is precedent for the use of the English FSSQ in the Tanzanian context [18].

The Patient Health Questionnaire-9 (PHQ9) survey, a widely validated screening tool for depression, was used to assess symptoms of depression among these patients (see Table 2). The survey has been previously translated to Swahili and validated in the East African region as well as in Tanzania specifically [19,20]. A socio-demographic questionnaire was used to record key patient variables (see Table 3).

**Table 2:** PHQ9 scoring system.

Numerical Score	Category of Depressive Symptoms
0–4	None-minimal depression
5–9	Mild depression
10–14	Moderate depression
15–19	Moderately severe depression
20–27	Severe depression

**Table 3:** Participant demographics.

	N (%)
**Age**	
15-30	22 (37)
31-50	21 (36)
50+	16 (27)
**Gender**	
Female	16 (27)
Male	43 (73)
**Marital Status**	
Married	30 (51)
Previously married	3 (5)
Never married	26 (44)
**Education**	
More than primary school	23 (39)
Primary school or less	35 (61)
**Region**	
Urban	15 (25)
Rural	44 (75)
**Religion**	
Catholic	19 (32)
Protestant	23 (39)
Muslim	14 (24)
Other	3 (5)
**Insurance status**	
Uninsured	47 (80)
Insured	12 (20)
**Injury location**	
Lower extremity	37 (63)
Upper extremity	12 (20)
Head	4 (7)
Torso	15 (25)
**Length of stay (LOS)**	
Short stay (6–11 days)	19 (32)
Medium stay (12–34 days)	15 (25)
Long stay (>34 days)	16 (27)

### 2.3 Statistical analyses

R software (Version 1.0.153) was used to conduct all quantitative analysis. Linear regression was used to measure the association between continuous outcomes (maladaptive coping score, adaptive coping score, functional social support score, PHQ9 total score) and participant characteristics in univariable analyses. 95% confidence intervals that do not span the null of 0 for continuous variables and null of 1 for ratios were considered statistically significant. The Fisher's Exact test was used to test the association between categorical variables and a p-value <0.05 was considered significant for this test. This work has been reported in line with the STROBE statement for cross-sectional studies [21].

## 3 Results

Of the 59 patients surveyed, 16 (27%) of the patients were female. The average age of the patients was 39 years old (range 15–80 years). Given this wide range, age was further divided into three tertiles: 15 to 30 (N = 22, 37%), 31 to 50 (N = 21, 36%) and older than 50 (N = 16, 27%). Patients surveyed were recovering from injuries to the upper extremity (20%), head (6·7%), torso (25·4%), and lower extremity (63%). Many patients were classified as polytrauma patients and suffered from injuries to multiple regions of the body. The average duration of treatment for the surveyed patients was 63 days with a range of six days to 919 days. Due to this wide range, tertiles of hospital-stay duration were used to create three categories: short stays (6–11 days), medium stays (12–34 days) and long stays (>34 days). Additional patient characteristics can be found in Table 3.

### 3.1 Coping strategies

54 (92%) of the 59 patients had an adaptive coping score that was greater than their maladaptive coping score. We found that a musculoskeletal injury to a patient's torso (i.e. chest or spine injury) was associated with a statistically significant increase in the degree of maladaptive coping strategies utilized (95% CI = 0·25,4·01) [Table 4]. Patients who were categorized as having long LOS (greater than 34 days) were found to use maladaptive coping strategies to a greater degree than those with a shorter LOS, though this finding narrowly missed the criteria for significance (95% CI = -0·04,4·10). This trend was reinforced by the fact that patients with long LOS also seemed to have a decreased use of adaptive coping strategies than those with short or medium LOS, although this was not statistically significant. The average Cronbach's alpha (measure of internal validity) for the coping strategies assessed using our survey was 0.43.

**Table 4:** Analysis of adaptive and maladaptive coping among participants.

	Adaptive Coping Score β coefficient (95% CI)	Maladaptive Coping Score β coefficient (95% CI)
**Age**		
15-30	–	–
31-50	0·29 (−2·68, 3·26)	0·38 (−1·55,2·31)
50+	−0·22 (−3·42,2·99)	−1·19 (-3·27,0·89)
**Gender**		
Female	–	–
Male	−1·36 (-4·16, 1·45)	0·69 (−1·18,2·55)
**Marital Status**		
Married	–	–
Previously married	0·77 (−5·13,6·66)	−3·27 (−7·16,0·43)
Never married	−0·31 (−2·92,2·30)	−0·87 (−2·54,0·81)
**Education**		
More than primary school	–	–
Primary school or less	−1·10 (−3·68,1·48)	−0·62 (−2·33,1·09)
**Region**		
Urban	–	–
Rural	−1·07 (−3·94,1·81)	0·02 (−1·90,1·93)
**Religion**		
Catholic	–	–
Protestant	−0·04 (−3·00, 2·92)	0·52 (−1·48, 2·52)
Muslim	−1·45 (−4·82, 1·92)	1·17 (−1·11, 3·44)
Other	−4·74 (−10·68, 1·21)	1·40 (−2·60, 5·41)
**Insurance status**		
Uninsured	–	–
Insured	2·45 (−0·61, 5·50)	0·94 (−2·15, 1·99)
**Injury location**		
Lower extremity	–	–
Upper extremity	−0·38 (−3·53, 2·76)	−0·46 (−2·52, 1·60)
Head	3·51 (−1·42, 8·44)	−1·23 (−4·46, 2·00)
Torso	−2·39 (−5·27, 0·48)	2·13 (0·25, 4·01)^a^
**Length of stay (LOS)**		
Short (6–11 days)	–	–
Medium (12–34 days)	−0·12 (−3·35, 3·11)	0·24 (−1·86, 2·35)
Long (>34 days)	−1·43 (−4·60, 1·74)	2·03 (−0·04, 4·10)^b^

a Indicates statistical significance.

b p-value = 0.054.

### 3.2 Functional social support

Patients with head injuries were found to have significantly more functional social support (95% CI = 0·97,6·63) Table 5. Several notable trends were also found. Patients without insurance were found to have less social support than those with insurance, though this trend was not significant (95% CI = 0·21,3·38). Those with less than a primary school education were also found to have a trend towards decreased social support (95% CI = -2·76,0·25). Lastly, being from a rural area rather than an urban area tended to result in less functional social support (95% CI = -2·73,0·63). The Cronbach's alpha for this section of our questionnaire was 0.47 for affective support and 0.58 for confidant support.

**Table 5:** Analysis of functional social support survey results.

	Functional Social Support Score β coefficient (95% CI)
**Age**	
15-30	–
31-50	0·44 (−1·3,2·17)
50+	−0·68 (−2·55,1·18)
**Gender**	
Female	–
Male	0·3 (−1·37,1·96)
**Marital Status**	
Married	–
Previously married	0·03 (−3·44,3·51)
Never married	0·42 (−1·12,1·95)
**Education**	
More than primary school	–
Primary school or less	−1·26 (−2·76,0·25)
**Region**	
Urban	–
Rural	−1·05 (−2·73,0·63)
**Religion**	
Catholic	–
Protestant	−0·14 (−1·94,1·66)
Muslim	−0·32 (−2·36,1·73)
Other	−0·65 (−4·25,2·95)
**Insurance status**	
Uninsured	–
Insured	1·59 (−0·21,3·38)
**Injury location**	
Lower extremity	–
Upper extremity	0·97 (−0·83,2·78)
Head	3·8 (0·97,6·63)^a^
Torso	−0·47 (−2·12,1·18)
**Length of stay (LOS)**	
Short (6–11 days)	–
Medium (12–34 days)	0·38 (−1·63,2·38)
Long (>34 days)	−0·16 (−2·13,1·81)

a Indicates statistical significance.

### 3.3 Depression

**Fig. 1:**
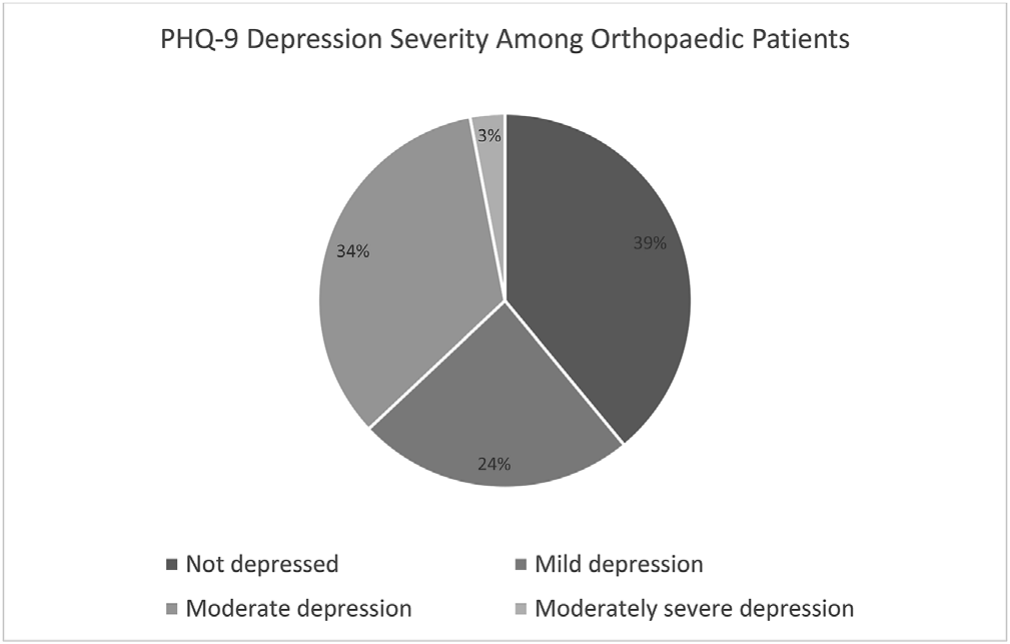
Distribution of depression severity among participants.

According to PHQ9 scoring protocol, 23·7% (14) of patients surveyed had symptoms consistent with mild depression, 33·9% (20) moderate depression, and 3.4% (2) moderately-severe depression Fig. 1. 23 (39%) patients displayed minimal to no depressive symptoms. The only significant predictor for a higher PHQ9 score was LOS. Patients who had long stays were found to have statistically significant increases in their PHQ9 score as compared to those who had short or medium stays (95% CI 1·59, 7·07)**.** Patients with long LOS (>34 days) had a 5·82 greater odds of having more severe depressive symptoms than other patients Table 6. Additionally, patients with short LOS (6–11 days) were three times less likely to have symptoms consistent with a more severe depression category than those with medium or long stays (OR 0·33; 95% CI = 0·11, 0·95). This trend of longer stays resulting in more severe symptoms was supported by the fact that patients with medium stays had higher PHQ9 scores than those with short stays, though this finding was not significant (95% CI = -1·28,4·3). The Fisher's exact test also showed a statistically significant association between a short LOS and the patient's PHQ9 depression category (p = 0·0009). The Cronbach's alpha for the PHQ9 total score in our patient cohort was 0.81.

**Table 6:** Analysis of PHQ9 depression screening scores of participants.

	PHQ9 Screening Score β coefficient (95% CI)	PHQ9 Screening Category Odds Ratio (95% CI)	PHQ9 Screening Category Fisher's Exact Test (P-value)
**Age**			
15-30	–	–	
31-50	0·59 (−2·03,3·21)	0·61 (0·26,1·42)	
50+	−0·91 (−3·73,1·92)	0·73 (0·32,1·64)	0·54
**Gender**			
Female	–	–	
Male	−0·6 (−3·12,1·91)	0·9 (0·32,2·53)	0·30
**Marital Status**			
Married	–	–	
Previously married	−0·53 (−5·67,4·61)	0·81 (0·09,7·02)	
Never married	−1·82 (−4·09,0·46)	0·63 (0·23,1·66)	0·58
**Education**			
More than primary school	–	–	
Primary school or less	1·2 (−1·07,3·48)	1·55 (0·58,4·23)	0·58
**Region**			
Urban	–	–	
Rural	0·86 (−1·7,3·42)	1·28 (0·44,3·79)	0·35
**Religion**			
Catholic	–	–	
Protestant	1·4 (−1·29,4·08)	2·31 (0·74,7·5)	
Muslim	1·17 (−1·88,4·22)	2·41 (0·68,8·85)	
Other	1·53 (−3·86,6·91)	3·87 (0·34,47·8)	0·64
**Insurance status**			
Uninsured	–	–	
Insured	−0·78 (−3·56,1·99)	0·62 (0·19,1·94)	0·07
**Injury location**			
Lower extremity	–	–	
Upper extremity	−0·61 (−3·47,2·25)	0·52 (0·13,1·9)	
Head	1·71 (−0·91,4·33)	1·85 (0·61,5·71)	
Torso	−1·06 (−5·55,3·43)	0·53 (0·06,3·4)	0.73
**Length of stay (LOS)**			
Short (6–11 days)	–	–	
Medium (12–34 days)	1·51 (−1·28,4·3)	1·71 (0·48,6·11)	
Long (>34 days)	4·33 (1·59,7·07)^a^	5·82 (1·59,23·44)^a^	0·001^a^

a Indicates statistical significance.

## 4 Discussion

Treatment of musculoskeletal injuries for patients in many LMICs is demanding physically as well as psychologically. The present study provides insight into the psychosocial health of patients recovering in the orthopaedic department of a tertiary referral hospital in Sub-Saharan Africa. This hospital serves as a referral center for millions of individuals in Northern Tanzania and has 630 official beds equipped for inpatient care. Symptoms of mild to moderately-severe depression are seen among 61% of patients receiving treatment in the orthopaedic surgery ward Fig. 1. This finding indicates that the majority of patients in the ward are in a state of psychiatric and emotional health that is suboptimal for successful clinical outcomes [7]. While KCMC is a tertiary hospital with relatively adequate resources to manage musculoskeletal injuries in comparison to surrounding medical centers, there is currently no psychiatry department or psychiatric services that can be offered to patients.

In addition, there is a lack of national resources devoted to the mental health needs of Tanzanians. As of 2014, there were approximately 134 mental health providers serving the 51 million citizens of Tanzania, only four of which practice outside the major city of Dar es Salaam [22]. The results reported here indicate that patients with musculoskeletal injuries have psychosocial concerns and they may benefit from mental health services. Previous studies have found that the rate of common mental disorders (including depression) in urban Tanzania is roughly 2·5% for males and 3·6% for females when using clinical interviews for diagnosis [23]. This indicates that the rate of depressive symptoms found in orthopaedic trauma patients far surpasses that of diagnosed depression in the general population. The high rate of depressive symptoms (61%) is also concerning in that it is roughly 5 times the rate of depressive symptoms among patients with musculoskeletal injuries in resource-rich countries such as Denmark [5].

Increased duration of stay was the most predictive factor for a high total PHQ9 score and a more severe depression category. The longer patients are kept in the hospital to receive treatment, the longer they remain separated from their daily routine, their jobs, and time with their families and communities. As reported by Davey et al. [24], 75·3% of orthopaedic patients at KCMC lost more than 30 days of functionality due to their injuries and 40·6% of patients lost their employment due to their injuries. These consequences can have devastating financial effects for patients and their families and contribute to the development of depressive symptoms. These symptoms appear to be accentuated by time, as patients spend weeks to months in bed recovering from their injuries. Increasing access to swift surgical care would decrease the LOS for many of these patients and potentially decrease the prevalence and severity of depression in this setting.

Patients with torso injuries, which often involved the spine, appear to have more severe symptoms of depression. Given the highly disabling nature of a spinal cord injury, it is possible that full-body immobilization and the associated complications (i.e. decubitus ulcers) could exacerbate or promote symptoms of depression. While spinal injuries are particularly debilitating, fractures of long bones in the lower extremities are by far the most common injury seen in this department [3]. Therefore, an increased ability to successfully treat even just lower extremity injuries surgically could result in significantly shorter hospitalizations, less psychological distress, and improved recovery for the population as a whole.

While rates of depression symptoms were high, nearly all patients in the cohort utilized adaptive coping strategies over maladaptive strategies to manage their injuries. Maladaptive coping strategies, such as denial or behavioral disengagement, could potentially interfere with successful treatment and rehabilitation. We speculate that maladaptive strategies such as substance abuse were not employed simply due to lack of access to substances during the patient's hospital admission. It would be interesting to examine if substance abuse became more prevalent as a coping strategy following discharge. In Moshi, Tanzania, it was found that 30% (n = 156) who presented to the hospital with injuries tested positive for alcohol in their system [25]. Therefore, it would also be helpful to determine if prior substance use is exacerbated in the recovery period. Again, disability appears to affect the degree to which maladaptive coping is employed by patients. Patients with torso injuries (i.e. chest wall or spinal injuries), who are likely the most disabled, were seen to utilize significantly more maladaptive coping strategies. This indicates that perhaps patients with torso injuries could benefit from additional services during hospital stays and attention from mental health professionals to modulate coping mechanisms.

Regarding functional social support, patients with cranial or cervical spine injuries had significantly more social support than others. This may be explained by the debilitating and disorienting nature of these injuries, which are often associated with sustained neurological deficits and the need for consistent support. It was also noted that being uninsured, having a primary school education or less, and having rural origins potentially decreases a patient's functional social support. These factors can all be considered markers of socioeconomic status and social network abundance. Those with a larger social network, perhaps from professional or scholarly connections, may have the privilege of having numerous strong social relationships to support them through a difficult recovery period. There may also be logistical barriers to social support, such as when a patient is from a remote rural area and his/her social network is far from the treatment center.

## 5 Limitations and future directions

The authors recognize that the combined patient survey used in this study is comprised of three previously validated surveys that have been translated to Swahili and modified, only one of which had been validated in Swahili [26]. Such changes would ordinarily warrant re-validation of the tool in the local context. However, given the logistical limitations placed on this project, such as time and expertise, it was not possible to formally validate the combined survey in the Tanzanian context. Given the compelling findings described here in this pilot study, formal validation of this survey in a larger population is certainly warranted. Validation would also increase the likelihood that the composite survey could be used to analyze psychosocial health in other clinical disciplines. In addition, the Cronbach's alpha values of internal validity may improve if the questionnaire is extended and further adapted to this Tanzanian context.

Another limitation of this study is the lack of a control group. As stated previously, prevalence data on common mental disorders (CMD), including depression, revealed a baseline rate of 2·5% in men and 3·6% in women in Tanzania [23]. However, these previous studies were conducted in urban settings which may not reflect the largely rural regions of Northern Tanzania. Another study on depression prevalence conducted in the Kilimanjaro region of Tanzania specifically looked at CMD risk among mothers of young children in this region [27]. Although this is a slightly different patient population, the rate of CMD, including depression, was found to be 28·8% in a sample size of 1922 participants. This percentage is still less than the 61% rate of mild to severe depressive symptoms found in our study, emphasizing the increased mental health needs of orthopaedic patients [27].

Only 59 patients met our inclusion criteria during the allotted time frame. This small sample size has inherent limitations. However, our sample size is comparable to similar studies aimed at understanding the psychosocial impact of musculoskeletal injuries and surgical care. A study of the orthopaedic trauma patient experience in Uganda consisted of semi-structured interviews with 35 patients admitted with a femur or tibia fracture [28]. Social support following repair of obstetric fistulas in Tanzania was also analyzed in a study with 28 participants [29]. Although these studies used different methodologies, this trend in sample size shows that our work is comparable to other studies conducted in the region. However, the generalizability of these results could certainly be enhanced by surveying more patients over a longer time frame.

Lastly, this study is also limited by the lack of treatment information for each specific patient. Patients with the same injury pattern but different treatment plans due to other barriers (i.e. patient's inability to pay or lack of material resources) may experience different mental health challenges. Given that the patients in our cohort stayed for at least six days and an average of 63 days, we know that they were likely treated conservatively with minimal surgical care. However, having an understanding of treatment specifics would be helpful to compare the mental health effects of specific treatment pathways. This deserves future study. Lastly, this study describes depressive symptoms of patients during inpatient treatment. Further work should be done to see if symptoms persist, worsen, or improve post-discharge due to common complications such as malunions, chronic pain, and/or deformity.

## 6 Conclusions

These results emphasize the mental and physical health benefits of improved surgical care and access in LMICs. As we improve the efficacy and efficiency of orthopaedic care in these settings, a large population of adults will experience less suffering and disability from musculoskeletal injuries. This research also affirms the need for mental health services in LMICs such as Tanzania, especially for patients with prolonged hospital stays for the treatment of musculoskeletal injury. Not only would quality of life be improved, but emotionally- and psychologically-well patients are more likely to achieve a successful clinical outcome. Given the relative scarcity of resources in these settings, creating an environment in which patients are more likely to benefit from the allocation of those resources is certainly ideal. It is essential that we see mental health as a crucial element of orthopaedic treatment in both high-resource and low-resource settings.

## Provenance and peer review

Not commissioned externally peer reviewed.

## Ethical approval

IRB approval from Kilimanjaro Christian Medical Center No. 2220, Proposal No. 1071.

## Sources of funding

Center for Global Health at the University of Pennsylvania, Student Travel Grant; The Dr. Bipinchandra Barahia Scholarship Fund.

## Author contribution

JEO conceptualized the study and performed the literature review. OS, HM, AP, FM, and RT contributed to local data collection and preliminary analysis. JEO conducted data analysis and data interpretation. AP and NPS contributed to data interpretation. JEO wrote the first draft of the manuscript with input from AP and NPS. AP, NPS, and EC critically reviewed and revised early drafts of the manuscript. All other authors critically reviewed drafts of the manuscript. All authors read and approved the final draft of the manuscript.

## Registration of research studies

Name of the registry: www.researchregistry.com.

Unique Identifying number or registration ID: researchregistry5007.

Hyperlink to the registration (must be publicly accessible): https://www.researchregistry.com/browse-the-registry#home/registrationdetails/5d24ee27d9459100102852fc/

## Guarantor

Joy E. Obayemi, BA.

## Declaration of competing interest

There are no conflicts of interest to report.
