# Supplementary material for: Psychosocial health of patients receiving orthopaedic treatment in northern Tanzania: A cross-sectional study
Source: Ann Med Surg (Lond). 2019 Nov 2;50:49–55. doi: 10.1016/j.amsu.2019.10.020 (PMC6994630; doi:10.1016/j.amsu.2019.10.020)
Supplement: Multimedia component 1 [file mmc1.docx]

**APPENDIX A:** Patient Survey (English)

A PSYCHOSOCIAL NEEDS ASSESSMENT OF PATIENTS RECOVERING FROM MUSKULOSKELETAL INJURIES AT KCMC

This survey is voluntary and anonymous. Your answers to this survey will not impact your care and are for research purposes only. Information about this survey and the research project can be found on the last page of the survey. You are welcome to take the last page of this survey home with you.

I understand this statement and am willing to participate in this research.

Signature:

**Basic Information**

**Mark the option that best describes you.**

1) Gender: ⬜ female ⬜ male

2) Age: __________

3) Marital Status: ⬜ single ⬜ married ⬜ divorced ⬜ widowed

4) Region: ⬜ Kilimanjaro

⬜ other (specify) ______________

5) District ⬜ Moshi Urban ⬜ Moshi Rural ⬜ Rombo ⬜ Siha

⬜ Same ⬜ Hai

⬜ Mwanga

⬜ other (specify) ______________

6) Village/Street: (specify)

____________________________________

7) What kind of area: ⬜ urban area ⬜ rural area

8) Tribe: ⬜ Chagga ⬜ Pare ⬜ Maasai ⬜ Sambaa

⬜ Meru ⬜ Iraq

⬜ other (specify)

9) Religion: ⬜ Catholic ⬜ Protestant ⬜ Seventh Day Adventist

⬜ Muslim ⬜ Hindu

⬜ Atheist

⬜ other (specify) ______________

10) Employment: (specify)

____________________________________

11) Education:

⬜ none ⬜ primary ⬜ secondary ⬜ high school

⬜ vocational ⬜ higher education

12) Monthly Income:

⬜ < 500,000 TSH

⬜ 500,000 - 1,000,000 TSH

⬜ 1,000,000 - 2,000,000 TSH

⬜ > 2,000,000 TSH

13) Date of admission ___________

**Injury Information**

14) Where is your injury located? ⬜ arm ⬜ elbow ⬜ wrist

⬜ hand

**Check all that apply**. ⬜ shoulder ⬜ leg above knee⬜ leg below knee⬜ hip/pelvis

⬜ knee ⬜ foot ⬜ ankle

⬜ back/spine

⬜ neck ⬜ face/head ⬜ chest

⬜ abdomen

⬜ urogenital

⬜ other ____________________

| **Health Insurance Information** |
| --- |

15) Do you have health insurance?

⬜ yes ⬜ no

If yes, which kind: _________________

**Coping Strategies Assessment^[[1]](#footnote-1)^**

These items deal with ways you've been coping with the stress in your life since you were injured.  There are many ways to try to deal with problems.  These items ask what you've been doing to cope with this one.  Obviously, different people deal with things in different ways, but I'm interested in how you've tried to deal with it.  Each item says something about a particular way of coping.   Try to rate each item separately in your mind from the others.  Make your answers as true FOR YOU as you can.

**1** = I haven't been doing this at all

**2** = I've been doing this a little bit 

**3** = I've been doing this a medium amount 
**4** = I've been doing this a lot

*Please circle the number that most accurately describes your experience.*

1.  I've been saying to myself "this isn't real."

1 2 3 4

2.  I've been concentrating my efforts on doing something about the situation I'm in.

1 2 3 4

3.  I've been using alcohol or other drugs to make myself feel better.

1 2 3 4

4.  I've been getting emotional support from others.

1 2 3 4

5.  I've been giving up trying to deal with it.

1 2 3 4

6.  I've been taking action to try to make the situation better.

1 2 3 4

7.  I've been refusing to believe that it has happened.

1 2 3 4

8.  I've been saying things to let my unpleasant feelings escape.

1 2 3 4

9.  I've been trying to see it in a different light, to make it seem more positive.

1 2 3 4

10.  I’ve been criticizing myself.

1 2 3 4

11.  I've been trying to come up with a strategy about what to do.

1 2 3 4

12.  I've been getting comfort and understanding from someone.

1 2 3 4

13.  I've been looking for something good in what is happening. 
 1 2 3 4

14.  I've been making jokes about it. 
 1 2 3 4

15.  I've been doing something to think about it less, such as reading, daydreaming, or sleeping.

1 2 3 4

16.  I've been accepting the reality of the fact that it has happened. 
 1 2 3 4

17.  I've been expressing my negative feelings. 
 1 2 3 4

18.  I've been trying to find comfort in my religion or spiritual beliefs. 
 1 2 3 4

19.  I’ve been trying to get advice or help from other people about what to do. 
 1 2 3 4

20.  I've been learning to live with my new situation. 
 1 2 3 4

21.  I’ve been blaming myself for things that happened. 
 1 2 3 4

22.  I've been praying or meditating. 
 1 2 3 4

23.  I've been making fun of the situation.

1 2 3 4

**Duke-UNC Functional Social Support Questionnaire (FSSQ)**

Here is a list of some things that other people do for us or give us that may be helpful or supportive. Please read each statement carefully and place an ‘X’ in the column that is closest to your situation. Give only 1 answer per row.

|  | As much as I would like | Almost as much as I would like | Some, but would like more | Less than I would like | Much less than I would like |
| --- | --- | --- | --- | --- | --- |
| 1. I have people who care what happens to me. |  |  |  |  |  |
| 2. I get chances to talk to someone about my problems |  |  |  |  |  |
| 3. I get a chance to talk about money matters |  |  |  |  |  |
| 4. I get useful advice about important things in life. |  |  |  |  |  |
| 5. I get help when I am sick. |  |  |  |  |  |

Source: adultmeducation.com

**Patient Health Questionnaire -9**

Over the last week, how often have you been bothered by any of the following problems? *(Please circle your answer)*

|  |    **Not at all**    | **Several days** | **More than half the days** |   **Nearly every day**   |
| --- | --- | --- | --- | --- |
| 1. Little interest or pleasure in doing things | **0** | **1** | **2** | **3** |
| 2. Feeling down depressed or hopeless |    **0**    | **1** | **2** |   **3**   |
| 3**.** Trouble falling or staying asleep, or sleeping too much | **0** | **1** | **2** | **3** |
| 4. Feeling tired or having little energy | **0** | **1** | **2** | **3** |
| 5**.** Poor appetite or overeating   | **0** | **1** | **2** | **3** |



| 6. Feeling bad about yourself — or that you are a failure or have let yourself or your family down |    **0** | **1** | **2** |   **3** |
| --- | --- | --- | --- | --- |
| 7. Trouble concentrating on things, such as reading the newspaper or watching television | **0** | **1** | **2** | **3** |
| 8**.** Moving or speaking so slowly that other people could have noticed? Or the opposite — being so fidgety or restless that you have been moving around a lot more than usual | **0** | **1** | **2** | **3** |
| 9**.** Thoughts that you would be better off dead or of hurting yourself in some way | **0**    | **1** | **2** | **3**   |

**If you checked off any problems, how difficult have these problems made it for you to do your work, take care of things at home, or get along with other people?** (*Circle your answer)*


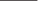

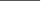


| Not difficult at all | Somewhat difficult | Very difficult | Extremely difficult |
| --- | --- | --- | --- |

Thank you for taking the time to complete this survey.

**APPENDIX B:** Patient Survey (Swahili)

**TATHMINI YA HALI AFYA KISAIKOLOJIA KWA WAGONJWA WENYE NAFUU WALIOPATA MAJERAHA YA MISULI NA MIFUPA HOSPITALI YA KCMC**

Utafiti huu ni wa hiyari na hautatajwa jina. Majibu yako hayatathiri ubora wa huduma yako au gharama zake na ni kwa ajili ya utafiti tuu. Uwe huru kuacha kuendelea na utafiti huu muda wowote pale unapoona inafaa. Kwa taarifa zaidi kuhusu utafiti huu tafadhali soma ukurasa wa mwisho. Na ukurasa huo unaruhusiwa kwenda nao nyumbani.

Kwa akili zangu timamu na bila kushurutishwa na mtu yeyote nimeelewa maelezo haya na niko tayari kushiriki kwenye utafiti huu.

Sahihi__________________________

| **Taarifa Muhimu** | |
| --- | --- |
| **Weka alama ya vema panapohusika.**    1) Jinsia: ⬜ KE ⬜ ME    2) Umri: _____________    3) Hali ya ndoa: ⬜ sijaoa/sijaolewa ⬜ ndoa ⬜ mtalaka ⬜ mjane    4) Mkoa: ⬜ Kilimanjaro  ⬜ mwingine (taja) ___________    5) Wiliya: ⬜ Moshi Mjini ⬜ Moshi Vijijini  ⬜ Rombo ⬜ Siha  ⬜ Same ⬜  Hai  ⬜ Mwanga  ⬜ mwingine (taja) ___________  6) Kijiji/Mtaa: (taja) ________________________    7) Unaishi wapi: ⬜ mjini ⬜ vijijini    8) Kabila: ⬜ Mchaga ⬜ Mpare  ⬜ Mmasai ⬜ Mmeru  ⬜ Msambaa ⬜ Muiraq  ⬜ nyingine (taja) _____________________________ | 9) Dini: ⬜ Mkatholiki ⬜ Mprotestanti  ⬜ Msabato ⬜ Muislam  ⬜ Mhindu ⬜ Mpagani  ⬜ nyingine (taja) ______________    10) Kazi: (taja)    ___________________________________________    11) Elimu: ⬜ hakuna  ⬜ shule ya msingi  ⬜ shule ya sekondari  ⬜ sekondari ya juu  ⬜ shule ya ufundi  ⬜ elimu ya juu    12) Kipato kwa Mwezi:  ⬜ < 500,000 TSH  ⬜ 500,000 – 1,000,000 TSH  ⬜ 1,000,000 – 2,000,000 TSH  ⬜ > 2,000,000 TSH    13) Date of Admission _____________________ |
| **Taarifa kuhusu Bima ya Afya** | |
| Je una bima ya afya? ⬜ ndio ⬜ hapana  Kama ndio, taja: _______________________________ | |
| **Taarifa za Jeraha / kuumia** | |
| Umeumia sehemu gani ya mwili? (**weka alama ya vema kila panapohusika**)  ⬜ mkono juu ya kiwiko ⬜ kiwiko ⬜ kifundo cha mkono ⬜ mkono chini ya kiwiko  ⬜ bega ⬜ paja ⬜ mguu chini ya goti ⬜ nyonga  ⬜ goti ⬜ unyayo ⬜ kifundo cha mguu ⬜ mgongo  ⬜ shingo ⬜ uso/kichwa ⬜ kifua ⬜ kiwiliwili  ⬜ sehemu za mfumo wa mkojo ⬜ nyingine (taja) ___________________________ | |

**Tathmini ya mkakati kwa mgonjwa katika kukubaliana na hali aliyonayo sasa**

Vitu vifuatavyo vinaelezea namna unavyopambana na msongo wa mawazo toka ulivyopata majeraha. Kuna njia nyingi za kutatua matatizo. Maswali haya yanakuuliza jinsi gani unatatua tatizo lako la sasa. Ni jambo lililo wazi kuwa watu hawawezi kutatua tatizo moja kwa njia inayofanana. Tumia machaguo ya namba hapo chini. Jaribu kufanya majibu yako yawe ya kweli kwa kadiri utakavyoweza.

**1** = Sifanyi kabisa 
**2** = Nimekuwa nikifanya kidogo sana

**3** = Nimekuwa nikifanya kwa wastani
**4** = Nimekuwa nikifanya sana

*Tafadhali zungushia duara namba inayoelezea uzoefu wako katika kukubaliana na hali uliyonayo.*

1. Nimekuwa nikijiambia "hali hii haijatokea kweli."

1 2 3 4

2.  Nimeweka jitihada za kutafuta suluhisho la tatizo nililonalo.

1 2 3 4

3.  Ninatumia kilevi au dawa nyingine ili nijisikie vizuri.

1 2 3 4

4. Nimekuwa nikipata msaada wa kihisia kutoka kwa wengine.

1 2 3 4

5. Nimekata tamaa ya kupambana na hali nilionayo.

1 2 3 4

6.  Nimekuwa nikijaribu kutafuta njia za kupata unafuu wa hali hii.

1 2 3 4

7.  Sitaki kuamini kama imetokea.

1 2 3 4

8.  Ninaongea vitu ili hisia hizi mbaya zipotee.

1 2 3 4

9. Nimekuwa nikijaribu kuona mazuri katika hali hii.

1 2 3 4

10. Nimekuwa nikijikosoa.

1 2 3 4

11. Nimekuwa nikijaribu kufikiria njia za kufanya.

1 2 3 4

12. Nimekuwa nikipata faraja na uelewa kutoka kwa mtu.

1 2 3 4

13. Nimekuwa nikitafuta sababu nzuri ya kwanini niliumia.
 1 2 3 4

14.  Nimekuwa nikifanya utani kuhusu hali yangu.
 1 2 3 4

15. Nimekuwa nikifanya vitu ili nisiwaze sana, kama kusoma au na kuwaza mambo mema.

1 2 3 4

16. Nimekuwa nikikubali hali halisi kwamba imeshatokea.
 1 2 3 4

17.  Nimekuwa nikionyesha hisia zangu hasi kuhusu hali yangu.
 1 2 3 4

18. Nimekuwa nikijaribu kutafuta faraja kwenye dini au imani yangu.
 1 2 3 4

19. Nimekuwa ninajaribu kupata ushauri au msaaada kwa watu ili kujua nifanye nini.
 1 2 3 4

20. Nimekuwa najifunza kuishi na hali hii.
 1 2 3 4

21. Nimekuwa nikijilaumu kwa vitu vilivyotokea.
 1 2 3 4

22. Nimekuwa nikisali au kutafakari.
 1 2 3 4

23. Nimekuwa sichukulii kwa uzito wa hali hii.

1 2 3 4

**Dodoso la Duke-UNC kuhusu msaada wa kijamii (FSSQ)**

Vifutavyo ni baadhi ya vitu ambavyo watu wengine wanafanya kwetu au wanatupatia ambavyo ni msaada kwetu. Tafadhali soma kwa makini na weka alama ya ‘X’ kwenye sehemu inayoelezea hali yako. Toa jibu moja tu.

|  | **Hapana kabisa** | **Kidogo** | **Kiasi** | **Sana** |
| --- | --- | --- | --- | --- |
| 1. Nina watu wanaonijali. |  |  |  |  |
| 2. Ninapata nafasi ya kuongea na mtu kuhusu matatizo |  |  |  |  |
| 3. Ninapata nafasi ya kuzungumzia mambo fedha. |  |  |  |  |
| 4. Ninapata ushauri kuhusu vitu muhimu katika maisha~~.~~ |  |  |  |  |
| 5. Ninapata msaada ninapokuwa mgonjwa. |  |  |  |  |

**KIDODOSI JUU YA AFYA YA MGONJWA -9**

Katika kipindi cha wiki moja zilizopita ni mara ngapi umesumbuliwa na matatizo haya yafuatayo? *(zungushia duara jibu linalofaa)*

|  | **Haijatoke zea kabisa** | **Siku kadhaa** | **Zaidi ya nusu ya siku hizo** | **Takriban kila siku** |
| --- | --- | --- | --- | --- |
| 1. Kutokuwa na hamu au raha ya kufanya kitu | **0** | **1** | **2** | **3** |
| 2. Kujisikia tabu sana au kukata tamaa | **0** | **1** | **2** | **3** |
| 3**.** Matatizo ya kupata usingizi au kuweza kulala au kulala sana | **0** | **1** | **2** | **3** |
| 4. Kujisikia kuchoka au kutokuwa na nguvu | **0** | **1** | **2** | **3** |
| 5**.** Kutokuwa na hamu ya kula au kula sana | **0** | **1** | **2** | **3** |
| 6**.** Kujisikia vibaya-au kujiona kuwa umeshindwa kabisa au umejiangusha au kuikatisha tamaa familia yako | **0** | **1** | **2** | **3** |
| 7. Matatizo ya kuwa makini kwa mfano unaposoma gazeti au kuangalia TV | **0** | **1** | **2** | **3** |
| 8**.** Kutembea au kuongea taratibu sana mpaka watu wakawa wameona tofauti? Au kinyume chake kwamba hutulizani na unahangaika sana kuliko ilivyo kawaida | **0** | **1** | **2** | **3** |
| 9**.** Mawazo kuwa ni afadhali zaidi ufe au ujidhuru kwa namna fulani | **0** | **1** | **2** | **3** |

**Kama ulitia alama matatizo yoyote, matatizo hayo yamefanya iwe vigumu kivipi kwako kufanya kazi yako, kushughulikia vitu nyumbani, au kutangamana na watu wengine?** (*zungushia duara jibu linalofaa)*

| Sio ngumu hata kidogo | Ngumu kiasi | Ngumu sana | Ngumu zaidi |
| --- | --- | --- | --- |

Asante kwa muda wako uliotumia kukamilisha utafiti huu

1. Carver, C. S.  (1997).  You want to measure coping but your protocol’s too long:  Consider the Brief COPE. *International Journal of Behavioral Medicine*, 4, 92-100. [↑](#footnote-ref-1)
